# Human Nasal Myiasis Caused by* Oestrus ovis* in the Highlands of Cusco, Peru: Report of a Case and Review of the Literature

**DOI:** 10.1155/2016/2456735

**Published:** 2016-12-26

**Authors:** P. Hoyer, R. R. Williams, M. Lopez, M. M. Cabada

**Affiliations:** ^1^School of Medicine, University of Texas Medical Branch, Galveston, TX, USA; ^2^Tropical Medicine Institute, Universidad Peruana Cayetano Heredia, Cusco Branch, Cusco, Peru; ^3^Infectious Disease Division, Department of Internal Medicine, University of Texas Medical Branch, Galveston, TX, USA

## Abstract

Myiasis is the infestation by dipterous larvae. The larvae can infect intact or decaying tissue including the skin or epithelial surfaces of the orbits, nose, and genitourinary and gastrointestinal tracts. We report a case of primary obligatory nasal myiasis by* Oestrus ovis* in a 56-year-old man from Cusco in Peru. He presented with nasal pruritus, congestion, and sneezing white “cottony” material. The material was identified as* O. ovis* larvae. A literature review of publications reporting nasal myiasis caused by* O. ovis* is presented.

## 1. Introduction 

Myiasis is a zoonotic infestation with dipterous larvae [1]. Biophagous larvae feed on living tissue in primary myiasis; however, secondary myiasis involves necrobiophagous larvae feeding on dead tissue [2]. Myiasis can be transmitted directly by deposition of eggs or larvae onto nonintact skin or mucous membranes. In addition, myiasis transmission can occur through a process called phoresis which begins when a female fly deposits eggs on arthropods that feed on mammals. When these egg carrying arthropods feed, the attached eggs hatch and burrow into the break in the skin created by the carrier [3]. In the case of* Oestrus ovis*, transmission occurs when females project larvae onto the muzzle (or face) of sheep (or humans) while flying. However, the accidental infestation by the introduction of larvae contaminated fingers into the nasal cavity has been proposed as an alternative route in humans [4]. Myiasis can be classified as facultative or obligatory. In obligatory myiasis, the infestation of viable tissue is necessary to complete the fly's lifecycle. In contrast, larvae in facultative myiasis may feed from necrotic tissues in the host but can also complete their lifecycle in the environment [5]. Cutaneous myiasis is the most prevalent form and furuncular lesions are a relatively common dermatological condition reported in travelers returning from South America and Africa [3, 6]. Less commonly, infestation can occur in body cavities with mucosal surfaces including the eyes, nose, and mouth. The most common presentation of* Oestrus ovis* in humans is ophthalmomyiasis which is an infestation of the soft tissue in the orbit [7]. Cavitary myiasis caused by* O. ovis* is rare in humans. However, in some countries, like Libya, the annual incidence of cavitary infestation may reach 1/10,000 among humans [8]. Other less common presentations of* O. ovis* can involve the oral cavity, pharynx, and tonsils. Humans in close contact with livestock, sheep in particular, are at higher risk to become accidental hosts for the* O. ovis* larvae. We present a case of* O. ovis* nasal myiasis in an adult patient from the highlands of Cusco, Peru.

## 2. Case Presentation

A 56-year-old male presented to his physician in the highlands of Cusco, Peru, complaining of two months of nasal pruritus and sensation of congestion, associated with sanguinolent discharge from his left nostril after waking up in the morning. He also reported having a burning sensation in his scalp and frequent sneezing after which white “cottony” material was expelled from his left nostril a few times in the previous couple of weeks. He managed to collect some of the material and brought it for analysis. The patient denied having fever, chills, headache, diplopia, blurred vision, halitosis, or neck stiffness. He had an injury to his nose over ten years before presentation with significant deformity of the septum. He had a history of heavy drinking and worked as a carpenter. The patient lives in Cusco city and denied owning livestock but admitted visiting rural areas where cattle and sheep are raised. On physical exam, the nasal septum was deviated to the left and the nasal mucosa was erythematous and friable and bled easily in the left nostril. There were no lymphadenopathies in the head or neck and no pain upon palpation of the sinuses. The rest of the physical exam was unremarkable.

**Figure 1:**
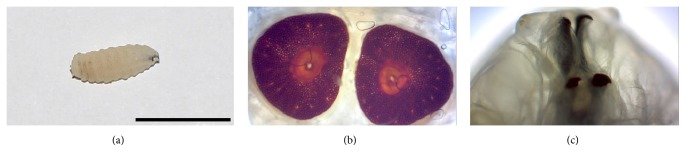
(a) Dorsal view of the* O. ovis* larva recovered by the patient (Bar = 10 mm). (b) Dark posterior spiracles with a flat side medially and respiratory holes arranged radially (40x). (c) Anterior hooks (40x).

**Figure 2:**
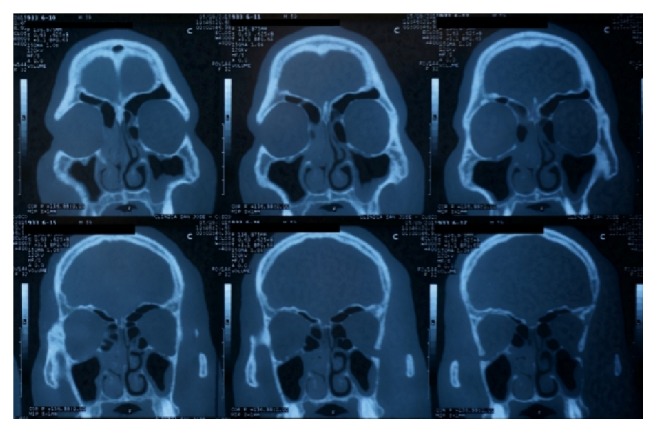
Coronal CT demonstrating mucosal thickening in the right nasal turbinates and left maxillary sinus.

The microscopic examination of the specimen brought by the patient revealed ~10 mm larvae with features compatible with* O. ovis*. The specimen demonstrated anterior hooks, dark posterior spiracles with a flat side medially, and respiratory holes arranged radially [Figure 1]. A computed tomography (CT) scan revealed significant mucosal edema in the right nasal cavity and left maxillary sinus [Figure 2]. Endoscopic examination of the nasal cavity and maxillary sinuses failed to reveal additional larvae. The patient was treated with a course of decongestants and remained in close follow-up. No further larvae were eliminated.

## 3. Discussion

*O. ovis* infestation is most common in shepherds in rural areas around the world but sporadic cases in nonshepherds have also been reported in recent years [15]. Although the most common hosts are sheep and goats, in the Cusco area* O. ovis *has been described in llamas [16, 17]. Thus, patients in these areas may not have a history of contact with sheep but may still be at risk of infestation. The most common presentation of* O. ovis* is ophthalmomyiasis. Infestation of the nasal cavity is an uncommon event in humans with only a few cases reported in the literature. Subjects with nasal myiasis due to* O. ovis* usually are middle aged men and women with a history of exposure to rural or tropical areas [Table 1]. Like in our patient, the nasal infestation is characterized by local inflammation of the mucosa and sneezing with spontaneous elimination of the larvae usually without leaving sequelae. All* oestrid* flies are host specific and only rarely infect accidental hosts [8]. The flies are larviparous and deposit their living offspring directly into or near the eyes and nose of sheep, their primary host [8]. The first instar (L1) larvae travel up into the nasal sinuses of their host and continue their maturation to the third instar (L3) stage. The L3 larvae drop from the nasal cavity to the ground for pupation [16, 18]. The duration of the life cycle, ranging from a couple weeks to several months, is largely dependent on the temperature and environmental conditions [16, 19]. Our patient presented with a long duration of symptoms which may have been related to the slow development of larvae in the cold environmental conditions from the highlands of Cusco.

Our patient was an accidental* O. ovis* host and probably became infested while being around sheep in rural areas of Cusco. Nasal myiasis is rarely complicated by erosion into adjacent structures like the orbit, nasal sinuses, and the palate [20].* O. ovis* infestation is considered a self-limited illness and, as in our patient, most cases reported have no evidence of extranasal compromise. However, the obstruction caused by the inflammatory reaction or the larvae themselves did result in a secondary sinus infection. The invasion of the intracranial space is an uncommon complication of nasal myiasis [5]. The presence of valveless veins that connect the orbit and nasal sinuses to the cranial fossa poses as a potential pathway for intracranial infestation [20]. However, only a few cases of intracranial myiasis or meningitis associated with myiasis have been reported, none of which involved* O. ovis* [3, 5]. In most of these cases there were predisposing conditions such as trauma or neoplasm that led to secondary facultative cerebral myiasis by species typically used for maggot therapy (*Calliphora vomitoria and Phaenicia sericata*) [3, 5].

In conclusion,* O. ovis* nasal myiasis is a rare occurrence in humans that most often causes a mild self-limited illness. Nonetheless, it can pose a diagnostic challenge to physicians unaware of this condition.

## Figures and Tables

**Table 1:** Human cases of nasal myiasis caused by *Oestrus ovis* reported in the literature.

Author/year	Age	Sex	Country	Exposure	Larval stage
Quesada et al. 1990 [9]	36	Male	Spain	Vacation to rural area	L1

Lucientes et al. 1997 [10]	64	Male	Spain	Living in rural area	L3

Einer and Ellegård 2011 [11]	65	Male	Sweden	Vacation to Greece	L2

Mumcuoglu and Eliashar 2011 [7]	33	Female	Israel	Living in rural area	L3

Hummelen et al. 2011 [12]	47	Female	Netherlands	Vacation to Republic of Cabo Verde	L3

Sante Fernández et al. 2015 [13]	43	Female	Spain	Living in tropical area	L1

Sacca et al. 1965 [14]	17	Male	Italy	Shepherd	Unknown
	50	Male	Italy	Shepherd	Unknown
	32	Male	Italy	Shepherd	Unknown
	30	Male	Italy	Shepherd	Unknown
	15	Male	Italy	Shepherd	Unknown
	16	Male	Italy	Shepherd	Unknown
	50	Male	Italy	Shepherd	Unknown
